# Protective effects of selenium on acrylamide-induced neurotoxicity and hepatotoxicity in rats

**DOI:** 10.22038/ijbms.2021.55009.12331

**Published:** 2021-08

**Authors:** Mahboobeh Ghasemzadeh Rahbardar, Hadi Cheraghi Farmad, Hossein Hosseinzadeh, Soghra Mehri

**Affiliations:** 1 Pharmaceutical Research Center, Pharmaceutical Technology Institute, Mashhad University of Medical Sciences, Mashhad, Iran; 2 School of Pharmacy, Mashhad University of Medical Sciences, Mashhad, Iran; 3 Department of Pharmacodynamics and Toxicology, School of Pharmacy, Mashhad University of Medical Sciences, Mashhad, Iran

**Keywords:** Acrylamide, Apoptosis, Hepatotoxicity, Neurotoxicity, Oxidative stress, Selenium

## Abstract

**Objective(s):**

Acrylamide (ACR), has wide uses in different industries. ACR induced several toxicities including neurotoxicity and hepatotoxicity. The probable protective effects of selenium on ACR-induced neurotoxicity and hepatotoxicity in rats were evaluated.

**Materials and Methods:**

Male Wistar rats were studied for 11 days in 8 groups: 1. Control, 2. ACR (50 mg/kg, IP), 3-5. ACR+ selenium (0.2, 0.4, 0.6 mg/kg, IP), 6. ACR+ the most effective dose of selenium (0.6 mg/kg, IP) three days after ACR administration, 7. ACR+ vitamin E (200 mg/kg IP, every other day) 8. Selenium (0.6 mg/kg IP). Finally, behavioral tests were done. The levels of malondialdehyde (MDA), glutathione (GSH), Bcl-2, Bax and caspase 3 proteins in liver and cerebral cortex tissues were measured. Also, the amount of albumin, total protein, alanine transaminase (ALT) and aspartate transaminase (AST) enzymes were determined in serum.

**Results:**

ACR caused the severe motor impairment, increased MDA level and decreased GSH content, enhanced Bax/Bcl-2 ratio and caspase 3 proteins in brain and liver tissues. Besides, the level of AST was elevated while the total serum protein and albumin levels were decreased. Administration of selenium (0.6 mg/kg) (from the first day of the experiment and the third day) significantly recovered locomotor disorders, increased GSH content, and reduced MDA level. Also, selenium decreased Bax/Bcl-2 ratio and caspase 3 levels in brain and liver tissues.

**Conclusion:**

The oxidative stress and apoptosis pathways have important roles in neurotoxicity and hepatotoxicity of ACR. Selenium significantly reduced ACR-induced toxicity through inhibition of oxidative stress and apoptosis.

## Introduction

Acrylamide (ACR) is a water-soluble and very reactive compound having a formula of C3H5NO (1). It is used in several industeris including cosmetics, printing, wastewater treatment and textile industries (2). It is also broadly found in high-carbohydrate foods cooked at high temperatures such as fried and baked foods (3). It was suggested that the mean dietary consumption of ACR in adults range 0.3 – 0.6 µg/kg bw/day. Children and teenagers are at greater risk since they use more foods such as fried potatoes, potato chips, and biscuits (4). Because of the intense toxicity of ACR in humans and animals, plenty of attention has been paid over the past few decades to control ACR toxicity (5). Different studies showed that ACR induced neurotoxicity which affects both the central and peripheral nervous system (6, 7), hepatotoxicity (8) and carcinogenicity (9). 

The pattern of ACR neurotoxicity might be similar to the usual pathological impressions seen in neurodegenerative disorders including Alzheimer’s disease and Parkinson’s disease (10). Several investigations suggested that ACR-induced neurotoxicity was correlated with oxidative stress (7, 11). Also, it has been observed that ACR interferes in enzyme activities and redox status in rats (12).

ACR has strongly boosted the levels of malondialdehyde (MDA), while reduced glutathione (GSH), succinate dehydrogenase (SDH), catalase, ATPase, superoxide dismutase (SOD) and lactate dehydrogenase (LDH) levels in the liver (8). An imbalance between the production and removal of reactive oxygen species (ROS) increases oxidative stress which is related to some disorders including liver and brain damages (13). 

Moreover, some other investigations reported that long-term exposure to ACR caused mitochondria collapse and led to apoptosis in human astrocytoma cells (14). Also, ACR csused significant apoptosis in both central and peripheral nervous system (6, 7). In line with these studies, some more evidence confirmed the apoptotic property of ACR in a dose and time-dependent manner in the liver (15, 16). 

Also, it has been reported that exposure to ACR decreased serum zinc, selenium, cobalt and magnesium concentrations (17).

 Regarding the wide exposure to ACR, it seems that research on compounds that can inhibit ACR-induced toxicity is really necessary. 

Selenium, a vital micronutrient, is a well-known detoxifying agent (18). Selenium insufficiency in the body might result in various disorders such as chronic degenerative diseases (19), and neurological disorders (20). Besides, selenium supplementation can protect from several ailments including immune system dysfunction (21), neurodegenerative disease (22) and viral infections (23). Selenium is also known for its anti-oxidant property. It was shown that this micronutrient could restore the function of main anti-oxidant enzymes for instance thioredoxin reductase and glutathione peroxidase (24).

The liver is located somewhere between the intestinal absorption site and the systemic circulation. This organ plays a key role in the detoxification of xenobiotics, environmental pollutants and chemical drugs (25). Selenium as a strong anti-oxidant can recover the damages of liver tissues caused by drugs and toxins. Various studies have shown that selenium, as a potent anti-oxidant, can alleviate liver toxicity induced by oxidative damage of toxins and various substances such as arsenic and silver nanoparticles (26, 27). Furthermore, studies showed that selenium and zinc co-supplementation after nonalcoholic fatty liver disease progression modified histological and biochemical parameters in an experimental model on male Sprague Dawley rats (28). Besides, it revealed that selenium consumption reduced apoptosis protein factors of caspase 3 and cytochrome c and elevated cyclin D in the liver tissue of cadmium-exposed rats (29).

Also, selenium seems to play a key role in the function of the nervous system (30). It was observed that the administration of selenium had a notable neuroprotective effect on ischemia/ reperfusion injury in a rat model by reducing oxidative stress (31). Selenium therapy had effective clinical outcomes on neurodegenerative disorders such as Alzheimer’s disease-induced dementia (32). Also, selenium declined arsenic-induced behavioral derangements through its anti-oxidant, anti-inflammatory, and anti-apoptotic properties in rats (33).

Considering the role of oxidative stress and apoptosis pathway in ACR neurotoxicity and hepatotoxicity, as well as, the anti-oxidant and anti-apoptotic effects of selenium, this study was designed to evaluate the protective effect of selenium on ACR toxicity. For this purpose, behavioral index, oxidative stress and apoptosis pathways in both brain and liver tissues were considered. 

## Materials and Methods

***Chemicals***

ACR (C3H5NO, > 99% purity), thiobarbituric acid (TBA), potassium chloride (KCl), phosphoric acid, and 5, 5^′^-Dithiobis 2-nitrobenzoic acid (DTNB) were purchased from Merck Company, Germany. Sodium selenite was obtained from Sigma Company, (Germany). Polyvinylidene ﬂuoride (PVDF) membrane from Bio-Rad, USA.

***Animals***

Tests were carried out on adult male Wistar rats, weighing 240–250 g. Animals were housed on a 12-h alternating light-dark cycle at a temperature of 22 ± 2°C, in the animal room of the School of Pharmacy, Mashhad University of Medical Sciences, Iran. Food and water were available ad libitum. All Experiments were approved by the Animal Care and Use Committee of Mashhad University of Medical Sciences (922757) and conducted in accordance with the Internationally Accepted Principles for Animal Use and Care.

***Experimental design***

Toxicity in male rats was induced by 50 mg/kg ACR intraperitoneally (IP) injection for 11 days (34, 35). To investigate the protective effects of selenium on ACR-induced toxicity, doses of 0.2, 0.4 and 0.6 mg/kg were used IP (36, 37). It should be noted that all injections (ACR and selenium) were performed daily and at a specific time. Selenium doses were injected to rats half an hour before ACR injection.

 For this study animals were randomly assigned to the following groups (n = 6):

Group 1: Animals receiving normal saline (control group)

Group 2: Animals receiving ACR 50 mg/kg IP for 11 days (34, 35).

Group 3: Animals receiving ACR 50 mg/kg IP for 11 days + selenium at 0.2 mg/kg IP

Group 4: Animals receiving ACR 50 mg/kg IP for 11 days + selenium at 0.4 mg/kg IP

Group 5: Animals receiving ACR 50 mg/kg as IP for 11 days + selenium at 0.6 mg/kg IP (37).

Group 6: Animals receiving ACR 50 mg/kg IP for 11 days + selenium at 0.6 mg/kg from the third day 

Group 7: Animals receiving ACR 50 mg/kg as IP for

11 days + Vitamin E 200 mg/kg IP every other day (35).

Group 8: Animals receiving selenium at a dose of 0.6

mg/kg IP

***Gait score examination***

At the end of treatment duration, animals were placed in a transparent box and their movements were assessed for 3 min. Their movements were ranked as follows:

1: Walking is normal.

2: Steps and walking are affected partially (minor weakness of the lower limbs).

3: Steps and walking are moderately affected (moderate weakness of the lower limbs).

4: Steps and walking have been heavily affected and lower limb paralysis has been established (7, 38).

***Sample collection***

After the behavioral test, animals were sacrificed, their brain (cerebral cortex), and liver samples were immediately obtained and frozen in liquid nitrogen, and then the samples were kept at -80 °C. Additionally the serum samples were collected and kept at -80 °C until biochemical analysis.

***Measurement of biochemical markers***

Alanine and aspartate aminotransferases (ALT & AST), total serum content of protein and total albumin were measured by Pars Azmun kit (Iran) in the biochemistry laboratory.

***Determination of MDA in brain and liver tissues***

MDA is a marker of lipid peroxidation that reacts with TBA and produces pink color complex which has maximum absorption in 532 nm (39).

To determine the level of MDA in the liver and cerebral cortex tissues, the 10% homogenate in 1.15% KCl was prepared. Then 0.5 ml of homogenized tissue was mixed with 3 ml phosphoric acid 1% and 1 ml of 6% TBA. Then the tubes were boiled in 95°C boiling water bath for 45 min. After cooling, n-butanol (4 ml) was added to the mixture and vortex-mixed for 1 min. The mixture was centrifuged at 3000 g for 20 min. Finally, the absorbance of the organic layer was recorded at 532 nm using (Jenway 6105 UV/Vis, UK). The MDA level was expressed as nmol/g tissue (6, 39).

***Determination of GSH content in brain and liver tissues***

The GSH content in tissues was measured using the moron, *et al.* method with minor changes (40). For this purpose, the 10% tissue homogenate was prepared in the buffer (pH 7.4). Then the sample was mixed with 10% TCA and then centrifuged at 3000 g for 10 min. After that, 0.5 ml of the supernatant was mixed with 2.5 ml of phosphate buffer (pH=8) and DTNB. Finally, the absorbance of the sample was measured at 412 nm using (Jenway 6105 UV/Vis, UK). The content of GSH was expressed as nmol/g tissue (40).

***Western blot analysis***

Brain and liver tissues were homogenized in a lysis buffer containing 50 mM Tris-HCl (pH: 7.4), 2mM EDTA, 2mM EGTA, 10 mM NaF, 1mM sodium orthovanadate (Na3VO4), 10 mM β-glycerophosphate, 0.2% W/V sodium deoxycholate, 1 mM phenylmethylsulfonyl fluoride, and complete protease inhibitor cocktail (Sigma Aldrich, USA). Protein concentration in different samples was determined by the Bradford assay kit (Bio-Rad, USA). The samples were mixed with 2X SDS blue buffer, boiled for 5 min, aliquoted, and kept in the -80 °C freezer. 

To determine the level of different proteins, samples were electrophoresed on a 12% (for Bax and Bcl-2) or 15% (for caspase 3) SDS polyacrylamide gel and transferred to a PVDF membrane (BioRad, USA). 

The blots were blocked with 5% skimmed milk in Tris-buffered saline tween 20 (TBST) on a rocker for 2 h at room temperature. Then blots were washed three times with TBST and incubated 2 hr at room temperature with rabbit polyclonal antiserum against Bax (Cell Signaling #2772, 1:1000), rabbit monoclonal anti-serum against Bcl-2 (Cell Signaling #2870, 1: 1000), rabbit monoclonal anti-serum against caspase 3 (Cell Signaling #9665, 1: 1000), mouse monoclonal anti-serum against GAPDH (Abcam #AB8245, 1: 1000). After washing three times with TBST, blots were incubated with anti-rabbit IgG labeled with horseradish peroxidase (Cell Signaling, #7074, 1:3000) or anti-mouse IgG labeled with horseradish peroxidase (Cell Signaling, #7076, 1:3000) for 1.5 hr at room temperature. Enhanced chemiluminescence (Pierce, USA) was used to visualize the peroxidase-coated bands. The optical densities of bands were measured using Alliance 4.7 Gel doc (UK) and densitometric analysis for protein bands was performed using UVtec software (UK). The protein levels were normalized relative to the corresponding bands of GAPDH as the control protein. 

***Statistical analysis***

GraphPad Prism 6.0 (GraphPad Prism Software Inc., San Diego, CA, USA) was used for statistical analysis. Results were shown as mean ± SD in lipid peroxidation assay, GSH content test, and western blot analysis. Statistical comparisons in the mentioned tests were made using one-way ANOVA followed by the Tukey–Kramer test. P values less than 0.05 were considered to be statistically significant. For the behavioral tests (gait abnormalities), data were expressed as median with interquartile range for each group and statistical analysis was accomplished with nonparametric test Kruskal–Wallis followed by Dunn’s Multiple Comparison.

## Results

***Effect of selenium on ACR-induced gait abnormalities***

**Figure 1:**
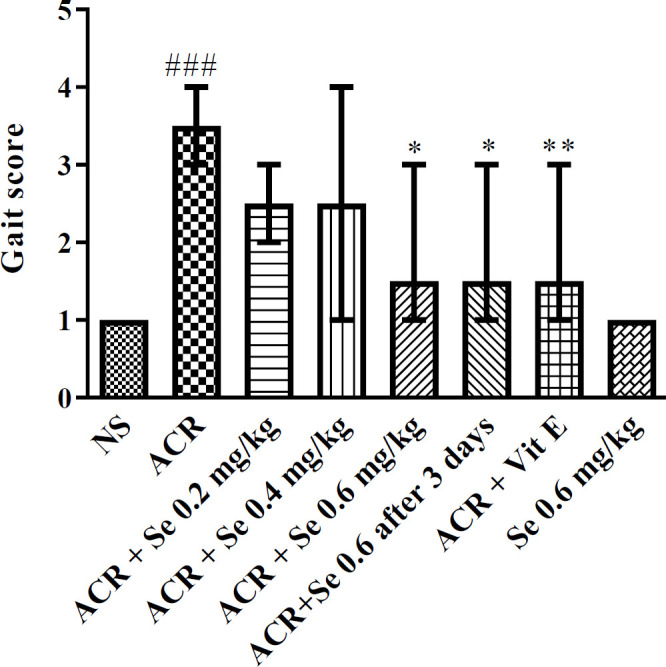
Effect of selenium on ACR-induced gait abnormalities. Data is expressed as median with interquartile range, (n=6). Statistical analysis was performed with nonparametric test Kruskal-Wallis followed by Dunn’s post test. ###*P*< 0.001 in comparison with control group and *P<0.05, **P<0.01 compared with ACR. NS: normal saline, Se: Selenium, ACR: Acrylamide, Vit E: Vitamin E

Administration of ACR 50 mg/kg for 11 days induced clear motor disturbances in rats compared to the control group (*P*<0.001). Administration of selenium at the dose of 0.6 mg/kg both simultaneously and also three days after starting the experiment could significantly reduce motor disorders induced by ACR (*P*<0.05) compared to the ACR group. Also, Vitamin E co-administration with ACR significantly reduced ACR induced disorders (*P*<0.01) in comparison with the ACR group (Figure 1).

***Effect of selenium on oxidative stress induced by ACR in the brain tissue***

**Figure 2:**
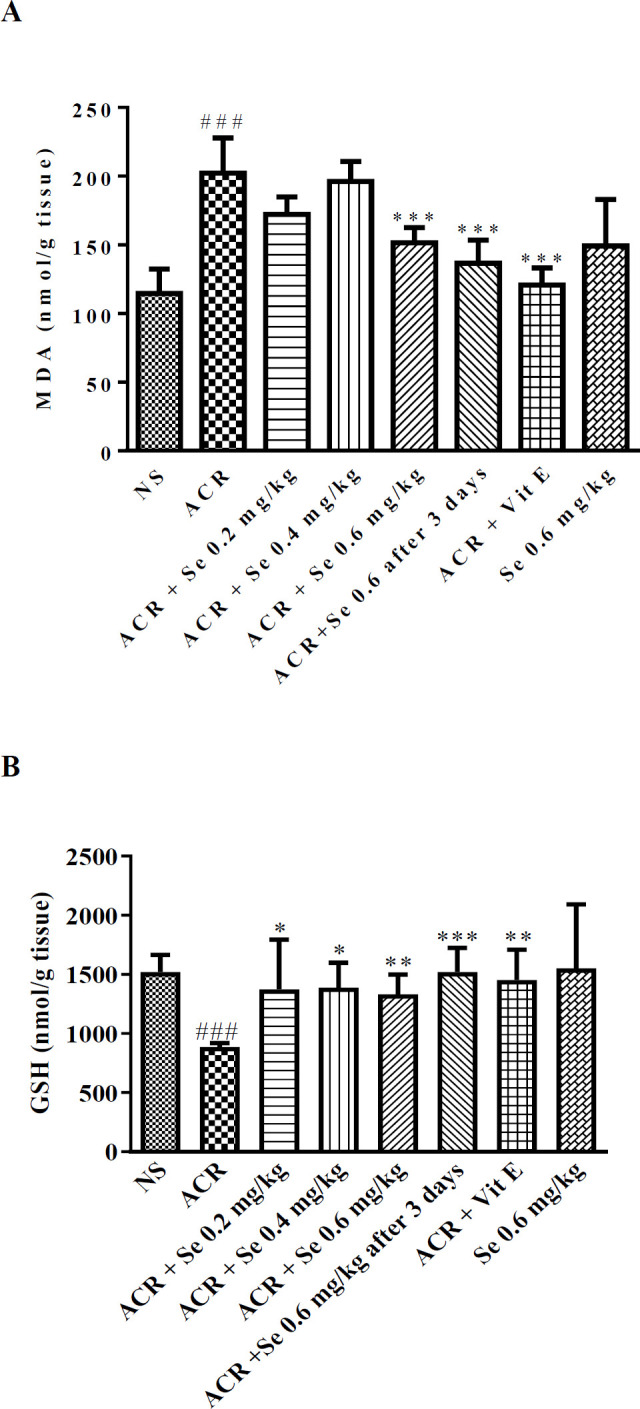
Effect of selenium on MDA level (A) and GSH content (B) in the cerebral cortex of ACR-treated rats. Data are expressed as mean ± SD (n=6). ANOVA and Post-test Tukey-Kramer were used for statistical analysis. ### *P*<0.001 compared with control group, and *P<0.05, **P<0.01 and ***P<0.001 compared with ACR group. NS: normal saline, Se: Selenium, ACR: Acrylamide, Vit E: Vitamin E

As shown in Fig 2A and 2B, the administration of ACR 50 mg/kg for 11 days caused a significant increase in the level of MDA and reduced the GSH content in the cerebral cortex compared to the control group (*P*<0.001). Selenium at 0.2 mg/kg dose (*P*<0.05), 0.4 mg/kg dose (*P*<0.05) markedly elevated the GSH content in comparison to ACR group. Also, selenium administration at dose 0.6 mg/kg (concurrent administration, or three days after ACR exposure) significantly increased the level of GSH and markedly attenuated the MDA level compared to the ACR group (*P*<0.001). Furthermore, vitamin E (200 mg/kg) co-administered with ACR, caused a considerable decline in the level of MDA (*P*<0.001) and produced a significant enhancement in the GSH content (*P*<0.01) when compared to the group which received ACR. 

***Effect of selenium on oxidative stress induced by ACR in the liver tissue***

**Figure 3:**
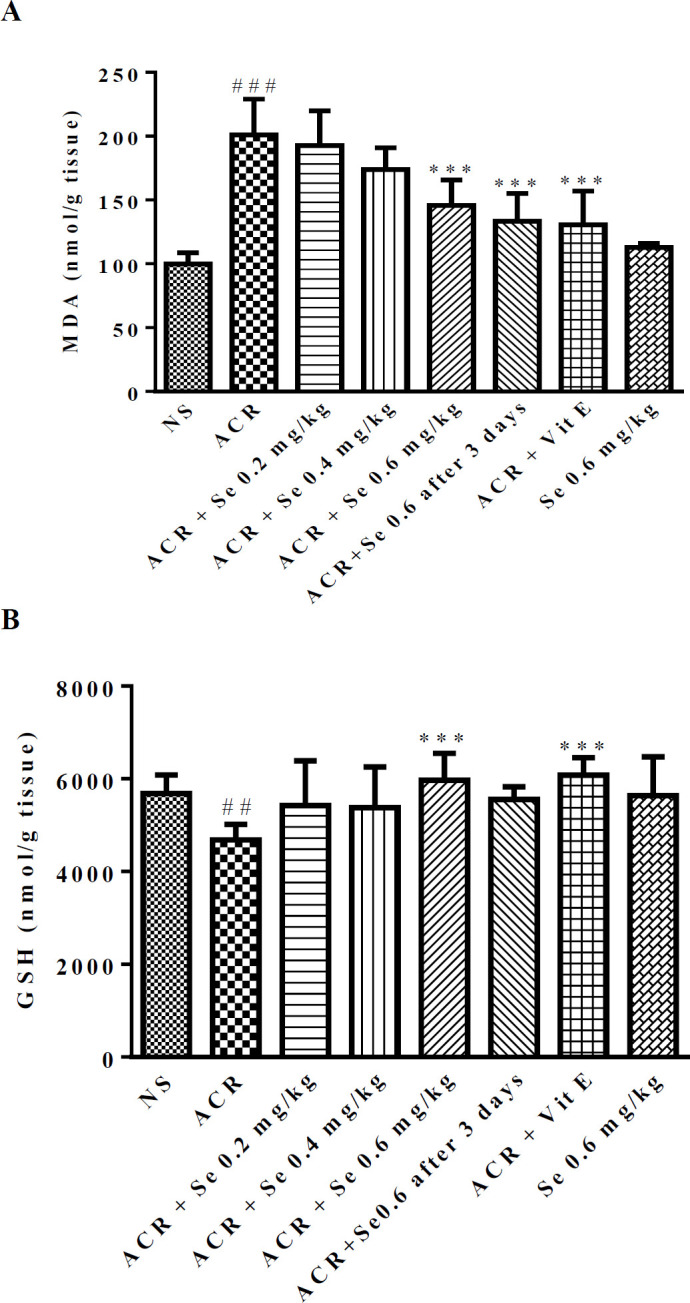
Effect of selenium on MDA level (A) and GSH content (B) in the liver of ACR-treated rats. Data are expressed as mean ± SD (n=6). ANOVA and Post-test Tukey-Kramer were used for statistical analysis. ### *P*<0.001, ##*P*<0.01 compared with control group, **P<0.01 and ***P<0.001 compared with ACR group. NS: normal saline, Se: Selenium, ACR: Acrylamide, Vit E: Vitamin E

The level of MDA was increased and the GSH content was reduced considerably in liver tissue following exposure to ACR in comparison to the control group. As indicated in Figure 3A, the administration of selenium at the dose of 0.6 mg/kg in both manners (simultaneously or three days after ACR exposure) could decrease the MDA level compared to the ACR group (*P*<0.001). Also, co-administration of selenium 0.6 mg/kg with ACR significantly reversed the GSH content compared to the group which received ACR (*P*<0.001) (Figure 3B). Concurrent administration of vitamin E with ACR markedly attenuated the level of MDA, while enhanced the GSH content when compared to the ACR group (*P*<0.001).

***Effect of ACR and selenium on serum albumin, total protein, ALT and AST levels***

Administration of ACR for 11 days increased the level of AST (*P*<0.01), decreased albumin (*P*<0.001) and total protein (*P*<0.05) in serum when compared to the control group. 

Exposure to ACR did not change the level of ALT in comparison with the control group. Administration of selenium at 0.2 and 0.4 mg/kg doses with ACR attenuated AST levels (*P*<0.05 vs ACR group). Administration of selenium at the dose of 0.6 mg/kg simultaneously and three days after administration of ACR increased total protein (*P*<0.05 and *P*<0.01 compared with ACR, respectively). Also, co-administration of vitamin E with ACR significantly decreased the AST level (*P*<0.05) and elevated albumin (*P*<0.01) and total protein (*P*<0.01) in serum compared to the group which received ACR (Table 1). 

***Effect of ACR and selenium on the level of proteins involved in the apoptosis pathway in the cerebral cortex tissue***

**Figure 4:**
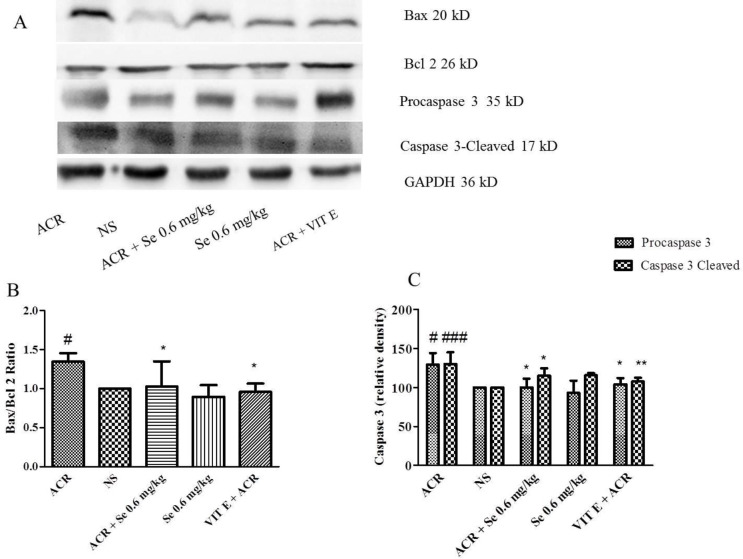
Effect of ACR and selenium on the level of proteins involved in apoptosis (Bax, Bcl-2 and caspase 3) in cerebral cortex tissue. A. specific bands for proteins involved in apoptosis (Bax, Bcl-2 and caspase 3), which have been investigated using Western Blot. B. Bax / Bcl-2 protein level data by densitometric analysis. C. Caspase 3 level data by densitometric analysis. Data are expressed as mean ± SD (n=4). ANOVA and Post-test Tukey-Kramer were used for statistical analysis. # *P*< 0.05, ### *P*< 0.001, compared with control group, *P<0.05 * and **P<0.01 compared with ACR group

Following administration of ACR, Bax/Bcl-2 ratio (*P*<0.05), procaspase 3 (*P*<0.05) and caspase 3-cleaved levels (*P*<0.001) increased in cerebral cortex tissue compared to the control group, while administration of selenium 0.6 mg/kg simultaneously with ACR reduced Bax/Bcl-2 ratio (*P*<0.05), caspase 3 (pro and cleaved forms) (*P*<0.05) level in comparison with ACR group (Figure 4).

It was also observed that co-administration of vitamin E with ACR reduced Bax/Bcl-2 ratio (*P*<0.05), procaspase 3 (*P*<0.05) and caspase 3-cleaved levels (*P*<0.001) in comparison to the group which received ACR.

***Effect of ACR and selenium on the level of proteins involved in the apoptosis pathway in the liver tissue***

**Figure 5:**
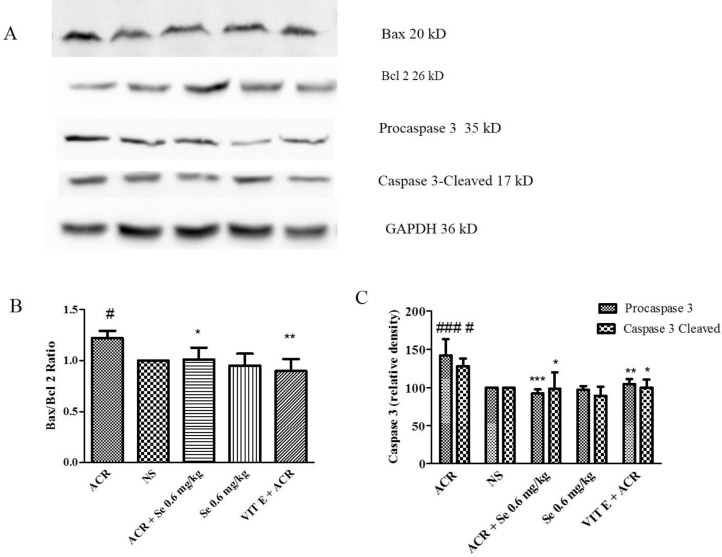
Effect of ACR and selenium on the level of proteins involved in apoptosis (Bax, Bcl-2 and caspase 3) in liver tissue. A. specific bands for proteins involved in apoptosis (Bax, Bcl-2 and caspase 3), which have been investigated using Western Blot. B. Bax / Bcl-2 protein level data by densitometric analysis. C. Caspase 3 level data by densitometric analysis. Data are expressed as mean ± SD (n=4). ANOVA and Post-test Tukey-Kramer were used for statistical analysis. #*P*< 0.05, ### *P*< 0.001 compared with control group, *P<0.05, **P<0.01 and ***P< 0.001 compared with ACR group

In the liver tissue, administration of ACR markedly elevated, Bax/Bcl-2 ratio (*P*<0.05 vs control group). Also, the level of procaspase 3 (*P*<0.001) and caspase 3-cleaved (*P*<0.05) was increased in the liver of ACR-treated animals in comparison to the control group. Co-administration of selenium (0.6 mg/ kg) with ACR significantly decreased apoptosis in the liver tissue which was confirmed with reduction in Bax/Bcl-2 ratio (*P*<0.05), procaspase 3 (*P*<0.001) and caspase 3-cleaved (*P*<0.05) levels compared with ACR group (Figure 5).

Additionally, the administration of vitamin E with ACR decreased Bax/Bcl-2 ratio (*P*<0.01), procaspase 3 (*P*<0.01), and caspase 3-cleaved levels (*P*<0.05) compared with the ACR group.

## Discussion

The protective effects of selenium on neurotoxicity and hepatotoxicity induced by ACR in rats were assessed in the current work. The obtained data revealed that ACR administration led to motor impairment, and induced oxidative stress and apoptosis in both brain and liver tissues. Administration of selenium (0.2, 0.4, and 0.6 mg/kg) for 11 days showed that the most effective dose was the highest dose (0.6 mg/kg) which could reverse the alterations induced by ACR. 

ACR is a classic toxicant formed in food, particularly in oven-baked and deep-fried foods (41). ACR has a neurotoxic effect on the nervous system in humans and animals and might result in various disorders including neurotoxic syndrome with determined hallmarks such as weight loss, skeletal muscle weakness, and ataxia (42). It has also been shown that ACR administration damages cellular macromolecules and degenerates neurons, which are the main causes of motor impairment (43). Moreover, several documents revealed that ACR causes hepatotoxicity by increasing oxidative stress, altering the function of liver biomarker enzymes (e.g. ALT, AST), as well as histological changes (44, 45). 

Hence, in this investigation, rats treated with ACR (50 mg/kg, IP, for 11 days), displayed paralysis in all their limbs. The intoxicated rats encountered weight loss and dragged their hind limbs. These effects of ACR were in line with another study which reported paralysis in 58% of the animals on day 10 due to exposure to ACR (46). On the other hand, rats receiving selenium (0.6 mg/kg) in both protocols (from the first day of the experiment and the third day), exhibited less paralysis in their limbs. 

The common tests to assess the function of the liver are measuring the amount of hepatic enzymes such as ALT and AST (47). Studies indicated that administration of ACR (25-50 mg/kg/day, PO) significantly increased AST, ALT levels and decreased serum albumin, and total protein amount in Wistar rats (48). Our data also displayed a reduced amount of albumin and total protein level in serum and increased content of AST and ALT after 11 days of ACR administration which reinforces the results of the previous studies. 

Selenium (0.6 mg/kg) co-administration could reverse ACR effect on the mentioned factors which support previous investigations in this field, including the study which reported that prescription of selenium (0.2 mg/kg, PO, 2 weeks) restores the reduced albumin level of peripheral blood mononuclear cells in streptozocin-induced diabetic mice (49). Moreover, another investigation reported that the administration of selenium nanoparticles (1.7 ppm, PO 30 days) and vitamin C declined ACR-induced elevation in ALT and AST levels besides decreasing the oxidative stress (50). 

Exposure to ACR can attenuate GSH content and elevate the MDA level and damage different tissues such as the brain and liver which shows an important role of oxidative stress in ACR-induced toxicity (35, 51). The administration of ACR in our study also revealed an elevation in the MDA level and a reduction in GSH levels in both tissues. Regarding to remarkable effects of selenium in different models of neurotoxicity, in the current research this element was selected as a neuroprotectice agent. Perviously effects of selenium in neurodegenartive diseases such as Alzheimer’s disease (32), ischemia/reperfusion injury (31), and arsenic-induced behavioral disorder (33), were considered. In our research, it was shown that the administration of selenium (0.6 mg/kg) from the first day of the experiment and the third day decreased the MDA level in brain and liver tissues in animals exposed to ACR. In addition, selenium (0.2, 0.4 and 0.6 mg/kg) administration concurrently with ACR increased GSH amount in brain tissue. But, in liver tissue, just the highest dose of selenium could increase the GSH level. In our study selenium significantly reduced ACR-induced neurotoxicity and hepatotoxicity through anti-oxidant properties. In line with our results, it has also been reported that the selenium prescription to lead-intoxicated animals ameliorated the activities of glutathione peroxidase, glutathione S-transferase, levels of MDA, and attenuated lipid peroxidation, regulated anti-oxidant defense system in different tissues including the liver and brain tissues (52). In another research, it was illustrated that selenium (1 mg/kg, PO 8 weeks) could reverse the MDA and GSH levels which were altered by ACR in the liver and kidney of rats (53). It was shown that in Huntington’s disease, selenium exhibited neuroprotective effects and prevents lipid peroxidation through increasing specific glutathione peroxidases (20). 

The activity of Bcl-2 and Bax proteins, the members of the Bcl-2 family, is conflicting. An increase in Bax, a pro-apoptotic protein, elevates the tissue apoptosis and an enhancement in Bcl-2 protein, anti-apoptosis protein, displays an anti-apoptotic activity. Accordingly, changes in Bax/Bcl-2 ratio determine the apoptosis level in tissue cells (54). Moreover, activation of caspases is a significant indicator of apoptosis (55). Different caspases activate during apoptosis, for instance, caspase 3 is a downstream effector and has an important role in the initiation of apoptosis (56). Apoptosis has been mentioned as another mechanism that is involved in ACR toxicity. It has been reported that oxidative stress and consequently mitochondrial/lysosomal damage and cell death signaling are important in ACR hepatotoxicity (15). Also, according to different studies, ACR-induced neurotoxicity can be mediated through the apoptosis signaling pathway (7, 57). Our obtained data also disclosed that after 11 days of ACR administration, the Bax/Bcl-2 ratio and the level of caspase 3 protein markedly elevated in both the cerebral cortex and liver tissues. Interestingly, prescribing selenium (0.6 mg/kg) exhibited an anti-apoptotic effect and reduced ACR hepatotoxicity and neurotoxicity through this pathway. The anti-apoptotic property of selenium plays important role in the hepatoprotective and neuroprotective effects of this micronutrient. It was observed that administration of selenium (1.5 mg/kg, twice a day, PO, 16 days) restricted apoptotic activity and histological alterations in cisplatin-induced neurotoxicity in rats (58). It also appeared that selenium (0.23 mg/kg, IP, 8 weeks) prescription has a direct anti-apoptotic property in the liver tissue of cadmium-exposed rats (29). In another research, oral administration of selenium (2 mg/kg, 90 days) reduced cadmium-induced apoptosis in chicken livers through reduction of Bax/Bcl-2 ratio and the level of caspase 3 protein (59).

In the current study, vitamin E was administered as a positive control because it has been reported to have hepatoprotective and neuroprotective effects mainly through anti-oxidant and anti-apoptotic properties against ACR-induced toxicity. Administeration of ACR significantly elevated the serum level of ALT, AST and ALP enzymes which declined following treatment with vitamin E. Also, vitamin E markedly reduced liver histological changes including lymphocytes infiltration, apoptosis, inflammation of portal space and fibrous expansion in some portal areas in mice which exposed to ACR (35, 60). 

**Table 1:** Effects of ACR and selenium on serum biochemical factors AST, ALT, albumin and total protein

**Total Protein** **(ng/ ml)**	**Albumin** **(** **ng / dl** **)**	**ALT** **(** **Units per liter** **)**	**AST** **(** **Units per liter** **)**	**Groups**
7.03± 0.3	4.13± 0.4	76.25± 8.01	213.8± 6.5	Control
5.77±0.7#	3.18±0.14 ###	94.20± 3.27	379 ± 129.9##	ACR
6.67± 0.7	3.4± 0.12	89.33± 1.035	254.5± 34. 89*	ACR + Selenium 0.2 mg / kg
6.68± 0.4	3.3±0.13	88.67±14.43	227± 2.64*	ACR + Selenium 0.4 mg / kg
6.69± 0.28*	3.4±0.1	81.1± 7.16	267± 17.16	ACR + Selenium 0.6 mg / kg
7.45± 0.44**	3.52± 0.19	88.6± 17.09	283.2± 20.02	ACR + Selenium (after 3 days) 0.6 mg / kg
6.9± 0.26	3.3±0.17	68± 6.9	228± 49.67	Selenium 0.6 mg / kg
7.07± 0.52*	3.88± 0.5**	76± 0.26	228.5± 46.99*	ACR + vitamin E

The data is expressed as Mean ± SD (n=6). ANOVA and Post-test Tukey-Kramer were used for statistical analysis. *P*< 0.001 ###, *P*< 0.01 ##, *P*< 0.05 # compared with control group, *P*<0.05 * and *P*<0.01 ** compared with ACR. Aspartate Aminotransferase (AST), Alanine Aminotransferase (ALT)

NS: normal saline, Se: Selenium, ACR: Acrylamide, Vit E: Vitamin E

## Conclusion

In brief, our data illustrate that ACR hepatotoxicity and neurotoxicity is originating from oxidative stress and ending in apoptosis and cell death signaling pathways. Selenium elevates GSH content and reduces lipid peroxidation in the liver and cerebral cortex. Also, selenium down-regulates proteins that are important in the apoptosis pathway. The anti-oxidant and anti-apoptosis effects of selenium are considered as main mechanisms in protection against ACR-induced toxicity.
